# Experiences with a simple laparoscopic gastric tube construction

**DOI:** 10.1186/1749-8090-8-14

**Published:** 2013-01-17

**Authors:** Bing-Yen Wang, Lien Cheng Tsao, Ching-Yuan Cheng, Ching-Hsiung Lin, Chih-Shiun Shih, Chia-Chuan Liu

**Affiliations:** 1 Department of Surgery, Changhua Christian Hospital, and School of Medicine, National Yang-Ming University, Taipei, Taiwan; 2 Institute of Medicine, Chung Shan Medical University, Taichung, Taiwan; 3 Division of Thoracic Surgery, Department of Surgery, Koo Foundation Sun Yat-Sen Cancer Center, Taipei, Taiwan

## Abstract

**Background:**

Minimally invasive esophagectomy (MIE) is a complex operation, and the detailed optimal surgical procedure has not been well described. Our aim was to evaluate use of a simple method of laparoscopic gastric tube construction as minimally invasive surgery for patients with esophageal cancer.

**Methods:**

We performed a retrospective review of 26 consecutive patients who underwent MIE for esophageal cancer in the Koo Foundation Sun Yat-Sen Cancer Center between September 2009 and August 2011. Perioperative data and postoperative complications were statistically analyzed.

**Results:**

The patient group consisted of 22 men and 4 women. MIE was performed successfully in all patients. The mean operative time was 430.4 ± 60.6 minutes, and the mean estimated operative blood loss was 135.0 ± 97.8 mL. There were no cases of conversion to open surgery during the procedure. The postoperative complication rate was 53.8%, and there was no surgical mortality.

**Conclusions:**

We recommend this novel method of total laparoscopic staplized formation of gastric tube to facilitate gastric pull-up.

## Background

Esophagectomy for esophageal cancer has remained the primary treatment for patients with localized disease. Numerous esophagectomy procedures have been described, but the surgery is technically complex and associated with high perioperative morbidity and mortality, even in high-volume hospitals [1,2]. However, with advances in endoscopic instruments and surgical techniques, esophagectomy has been increasingly performed by means of a minimally invasive approach. Luketich and colleagues [3] described a detailed method of total minimally invasive esophagectomy (MIE) and concluded that it could be performed with low morbidity and mortality rates. They performed total laparoscopic gastric staplized tube construction without mini-laparotomy. In the laparoscopic stage, the most cephalad portion of gastric tube must be attached to the esophageal specimen using 2–0 Endo-sutures. In addition, a superficial stitch may be placed on the anterior proximal gastric tube to facilitate orientation and prevent twisting as the tube pulled-up.

In this study, we modified the surgical procedures in the laparoscopic stage. We describe a novel and simple method for total laparoscopic staplized formation of gastric tube to facilitate gastric pull-up. Perioperative data and postoperative complications were statistically analyzed.

## Methods

### Patients

We retrospectively reviewed consecutive patients who underwent MIE for esophageal cancer at the Koo Foundation Sun Yat-Sen Cancer Center from September 2009 to August 2011. A total of 26 patients were enrolled in the study. All operations were performed by one surgeon. In our institution, MIE was adopted beginning in 2007. In September 2009, we developed a new simple method of laparoscopic gastric tube construction to facilitate gastric tube pull-up. Neoadjuvant chemoradiotherapy was offered to patients with esophageal cancer staged as T2 and higher. After neoadjuvant treatment, cases of stable disease or regressive tumor response were considered for esophagectomy. MIE was tried initially for every patient for whom esophagectomy was planned except those for whom colon interposition was scheduled to be performed.

All of the 26 patients received complete evaluation before neoadjuvant therapy and surgery, including physical examinations, biochemistry tests, upper gastrointestinal panendoscopy, endoscopic ultrasound, bronchoscopy, chest and abdominal computed tomography, and positron emission tomography–computed tomography. The detailed surgical technique has been previously described [4]. Briefly speaking, the operative method is composed of thoracoscopic esophageal mobilization followed by laparoscopic construction of the gastric tube and esophagogastric anastomosis in the neck. In the laparoscopic stage, five abdominal trocars are placed. The stomach was mobilized by dividing short gastric vessels, left gastroepiploic arcade and left gastric vessels. The gastric tube is formed from the distal aspect of the lesser curvature of the stomach with the use of multiple linear 3.8 mm endostaplers. The proximal, uppermost aspect of the lesser curvature near the esophago-cardiac junction is trimmed with a linear endostapler with preservation of the connection between the esophagus and the gastric tube. Under laparoscopic guidance the gastric conduit is pulled up to the neck incision thorough the hiatus. The key point of techniques was to preserve the esophago-cardiac junction, which is used to facilitate gastric tube pull-up. The method could avoid dividing the esophagocardiac junction and attached again by silk sutures.

Clinical data were collected, including age, sex, preoperative comorbidities, perioperative data, postoperative complications, length of hospital stay, and tumor characteristics. Surgical mortality was defined as death during the same hospitalization or within 30 days after the operation. Informed consent was obtained from each patient before treatment.

After MIE, temporary mechanical ventilator support was provided to most patients, and patients were weaned from the ventilator based on weaning parameters and the patient’s general condition. Feeding via jejunostomy was begun on the first postoperative day, and the amount was increased gradually. Esophagography was performed on the eighth postoperative day to evaluate the cervical anastomosis. Anastomotic leakage was defined as spillage of saliva or gastric contents found at the cervical wound or contrast extravasations by esophagophography. Pneumonia was defined as positive sputum cultures and chest radiographic evidence of consolidation. Hoarseness was defined as voice change after extubation. Epidural analgesia is no longer used in patients undergoing MIE in our institution, and its potential risk was therefore avoided.

### Pathologic examination

All specimens were sent for pathologic examination after preservation in 10% neutral buffered formalin. The depth of tumor invasion, grade of differentiation, and lymph node–involving status were recorded according to the results of pathologic reports. All patients were staged using the seventh edition of the American Joint Committee on Cancer tumor-nodes-metastasis staging system manual [5].

### Statistical analysis

Continuous data were expressed as mean ± standard deviation. Data analysis was performed using SPSS software (version 12.0; SPSS, Chicago, Ill).

## Results

The study participants consisted of 22 men and 4 women with a mean age of 54.7 years (range = 40–70 years). Detailed clinical characteristics of all patients are listed in Table 1. A total of 15 patients received preoperative chemoradiotherapy. Perioperative data are presented in Table 2. Thoracoscopic esophagectomy with laparoscopic gastric construction was completed in all 26 patients. There were no cases of conversion to open thoracotomy or laparotomy. Gastric conduit was used as a reconstruction substitute in all patients. The mean operative time was 430.4 ± 60.6 minutes, and the mean operative blood loss was 135.0 ± 97.8 mL. The mean length of hospital stay was 13.5 ± 9.1 days. Postoperative complications are shown in Table 3. There were a total of 18 complications in 14 patients. There were four cases of anastomotic leakage, which was treated by wet dressing without second operation. There was no surgical mortality and re-operation in this series.

**Table 1:** Patient Demographics (n = 26)

	**N**
Age, years, mean ± SD (range)	54.7 ± 8.6 (40–70)
Gender	
Male	22
Female	4
Comorbidities	
Hypertension	3
COPD	2
Chronic renal insufficiency	1
Tuberculosis	1
Liver disease	4
Previous malignancy	2
Peptic ulcer	3
Diabetes mellitus	3
Others	2
Neoadjuvant CCRT	
Yes	15
No	11

*CCRT* = concurrent chemoradiotherapy.

*COPD* = chronic obstructive pulmonary disease.

**Table 2:** Perioperative Data

**Variables**	**Value**
Operation time, mean ± SD (mins)	430.4 ±60.6
Blood loss, mean ± SD (ml)	135.0 ± 97.8
Ventilation day, mean ± SD (days)	1.5 ± 3.4
Length of ICU stay, mean ± SD (days)	3.2 ± 6.2
Length of hospital stay, mean ± SD (days)	13.5 ± 9.1
Conversion rate (%)	0
Complication rates (%)	53.8%
Surgical mortality (%)	0

*ICU* = intensive care unit.

*SD* = standard deviation.

**Table 3:** Postoperative Complications

**Complication**	**Number(%)**
Anastomotic leak	4(15.4)
Hoarseness	8(30.7)
Horner syndrome	1(3.8)
Pneumonia	1(3.8)
Atrial fibrillation	1(3.8)
Empyema	1(3.8)
Wound infection	1(3.8)
Respiratory failure	1(3.8)
Chylothorax	0(0)

Twenty-four patients (92.3%) in the study were diagnosed with squamous cell carcinoma. The mean number of lymph nodes included in the specimens was 37.2 ± 10.2. There were two cases of microscopic positive circumferential margins and no cases of proximal or distal positive cut end.

## Discussion

Since the development of thoracoscopic and laparoscopic surgery in the early 1990s, numerous reports have described different procedures for MIE. The various types of endoscopic surgery described have included thoracoscopic transthoracic esophagectomy, laparoscopic transhiatal esophagectomy, laparoscopic gastric preparation, and transcervical mediastinoscopy as well as combination, or hybrid, techniques [3,6,7]. However, no direct randomized clinical trial comparing the different surgical endoscopic methods has been conducted because of the wide variety of techniques and the relatively low number of procedures performed; therefore, the optimal surgical approach for esophageal cancer is still under debate. In addition, MIE has not yet gained widespread acceptance in the surgical community, mainly because of concerns about its surgical safety, technique difficulty, and steep learning curve. Therefore, no consensus has developed regarding the superiority of any particular MIE approach.

Luketich and colleagues [3] reported the largest single-institutional series of MIE procedures. The perioperative data confirmed that MIE without mini-laparotomy could be performed with low rates of complications (32%) and mortality (1.4%). The short-term postoperative results suggested that MIE is a safe and feasible surgical procedure in experienced hands. They also described the detailed surgical procedures, including thoracoscopic mobilization of the esophagus and laparoscopic gastric tube construction. However, most thoracic surgeons are not familiar with laparoscopic skills, especially in suturing and knotting, so they may require more cases to overcome the learning curve.

A similar operative technique was adopted in the present study, and we modified the method of total laparoscopic staplized gastric tube construction. Conventional gastric tube construction involves dividing the cardiac part and body part of the stomach and attaching the gastric tube to the esophageal specimen by silk sutures for gastric tube pull-up to the neck. Our modified gastric tube construction [4] may have several advantages over the conventional procedure. The sutures between the esophageal specimen and the gastric tube may rupture when the gastric tube is pulled up by the connection of the esophagus with sutures. We preserved the connection of the esophagus and the gastric tube, which was strong enough to avoid disconnection. Additionally, the traction force may be distributed more equally, which may avoid angulation of the gastric tube. Finally and most importantly, it is a simple method of gastric tube formation that avoids laparoscopic suturing and knotting and facilitates gastric tube pull-up.

The reported complication rate of esophagectomy ranges from 30% to 80%, depending on the criteria used to define complications. In this study, the morbidity rate was 53.8%, and there was no surgical mortality. There was no gastric tip necrosis or gastric tube ruption with the simple laparoscopic gastric tube construction. The most common postoperative complication was hoarseness (30.7%). In our study, the definition of hoarseness was voice change on the day of extubation. This voice change may be caused by transient postoperative vocal cord swelling. We did not evaluate vocal cord movement routinely after MIE. All the patients could eat smoothly without choking after discharge.

The optimal postoperative pain control methods for thoracoscopic and laparoscopic surgery have been controversial. Epidural anesthesia may be the most popular and well-known option, but several associated complications have been reported in the literature, including nausea, vomiting, hypotension, pruritus, constipation, and technical complications [8]. Thoracic epidural analgesia was not used in any of the 26 patients, avoiding its potential risk. We prescribed oral nonsteroidal anti-inflammatory drugs and oral opioids for postoperative pain control. Some patients needed several additional injections of intravenous opioids on the first postoperative day. To our knowledge, no study has yet addressed the efficacy of thoracic epidural analgesia in MIE. In our series, the rate of postoperative respiratory complications was low (one case of respiratory failure and one case of pneumonia), which may indicate that thoracic epidural analgesia may not be routinely administered in the postoperative care of MIE.

## Conclusions

Our retrospective analysis demonstrates the technical feasibility of simple laparoscopic gastric tube construction. It may facilitate MIE through safer staple application and easier gastric tube pull-up.

## Abbreviations

CCRT: Concurrent chemoradiotherapy; COPD: Chronic obstructive pulmonary disease; ICU: Intensive care unit; MIE: Minimally invasive esophagectomy; SD: Standard deviation.

## Misc

Bing-Yen Wang and Lien Cheng Tsao are contributed equally to this work

## Competing interests

The authors declare that they have no competing interests.

## Authors’ contributions

CCL and BYW performed the operations and wrote the manuscript. LCT and CYC collected the clinical data. CHL and CSS provdied the technical support. All authors read and approved the final manuscript
